# Predictors of Vitamin C Status in National Health and Nutrition Examination Survey 2017–2018: The Effect of Body Weight and Body Mass Index in a Multiple Fractional Polynomial Analysis

**DOI:** 10.1016/j.tjnut.2025.10.001

**Published:** 2025-10-08

**Authors:** Julia K Bird, Edith JM Feskens, Alida Melse-Boonstra

**Affiliations:** Division of Human Nutrition and Health, Wageningen University and Research, Wageningen, The Netherlands

**Keywords:** vitamin C, dietary reference intakes, obesity, BMI, NHANES

## Abstract

**Background:**

This study aims to set vitamin C requirements for all age, sex, and life cycle groups. Requirements calculated for healthy young males are extrapolated based on body weight. Increasing obesity prevalence in recent decades has led to higher body weights in many countries; hence, this may increase vitamin C requirements.

**Objectives:**

This study aims to assess the relationship between body weight/obesity, and other covariates, associated with serum vitamin C status.

**Methods:**

Data from adult participants of the cross-sectional survey National Health and Nutrition Examination Survey 2017–2018 (*N* = 4556) were used. The multiple fractional polynomial method was used to quantify linear or nonlinear associations between vitamin C status and obesity, including relevant covariates identified from the literature. The covariates included were gender, smoking, C-reactive protein (CRP), serum albumin, recent illness, physical activity, and vitamin C intake.

**Results:**

There was a positive, linear relationship found between serum vitamin C, and serum albumin and physical activity. There was a negative, linear relationship between serum vitamin C, and bodyweight or body mass index, and a weak U-shaped relationship with age. Serum vitamin C and vitamin C intake showed a positive, nonlinear relationship, whereas there was a negative, nonlinear relationship between vitamin C status and CRP. Vitamin C status was lower in males and in people with higher smoking exposure. Recent illness was not associated with vitamin C status.

**Conclusions:**

These results in a large, representative dataset indicate that greater body weight or obesity may increase vitamin C requirements. In addition, nonlinear associations such as between vitamin C intake and status should be taken into consideration when determining requirements.

## Introduction

An adequate intake of vitamin C is required to prevent vitamin C deficiency and its consequences. Symptoms of vitamin C deficiency include loss of appetite, fatigue and lethargy, bruising and bleeding gums, and may progress to scurvy if vitamin C intakes are minimal for several weeks [1]. Low vitamin C status and deficiency are relatively common in resource-poor countries due to inadequate intakes. In high-income countries, certain population groups are at greater risk of low vitamin C status due to lifestyle, chronic disease, and genetic factors [2]. Two repeated cross-sectional analyses of vitamin C status in the United States population found that mean serum vitamin C status was stable between 2005–2006 and 2017–2018, with a mean reported of 51.2 and 54.0 μmol/L, and a weighted prevalence of vitamin C deficiency (serum vitamin C <11.4 μmol/L) of 7.0 % and 6.8%, respectively [3].

There has been recent interest in the effect of increasing obesity prevalence, and the resulting increase in body weight, on vitamin C status [[4], [5], [6]]. Dietary intake recommendations use isometric scaling by body weight to derive reference intakes for females and children from pharmacokinetic studies performed in healthy young males [1,7,8]. Using the same rationale employed in deriving the dietary intake recommendations, the increase in body weight seen in many countries in recent decades may render a broader proportion of the population at risk of low vitamin C status. In support of this theory, many epidemiological studies have found that obese individuals have a lower vitamin C status [9]. Limited evidence from an intervention study also supports this relationship. In a clinical study performed in subjects given the same supplemental vitamin C dose showed that higher body weight led to a blunted plasma vitamin C response to supplementation, although intersubject variability was high [10]. In this depletion–repletion study, subjects in the highest body weight quartile (91 kg) reached a plasma vitamin C concentration that was ∼10 μmol/L lower than subjects in the lowest body weight quartile (59 kg) during 4 wk of repletion at 117 mg/d. Nevertheless, one-third of subjects did not reach a plasma plateau during this repletion period, and the blunted response to supplementation in heavier subjects may be responsible.

In addition to higher body weight, other factors are present in individuals that may affect vitamin C status. Nutrient partitioning may dampen the effect of body weight on vitamin C status. Indeed, the greater proportion of lean mass is suggested to be a factor in the lower vitamin C status found in males [1]; even so, a strong correlation between plasma and muscle vitamin C has been found [11]. Potentially, there is a higher turnover of vitamin C in muscle. Although the vitamin C content of adipose tissue is not generally included in models of whole-body vitamin C homeostasis [12], the high fat content of adipose tissue may have a lower relative concentration of water-soluble components, including ascorbic acid. Thus, the effect of additional body weight in individuals with obesity on vitamin C concentrations might be less pronounced than expected. Nevertheless, some evidence supports higher adiposity affecting vitamin C status through alternative mechanisms such as reduced absorption, increased urinary excretion, or enhanced turnover through systemic inflammation [5].

In terms of lifestyle factors, cigarette smoking is known to reduce vitamin C status by increasing turnover. Smokers’ lower vitamin C status and higher prevalence of vitamin C deficiency were found in 2 representative samples of the United States population taken in 2003–2006 and 2017–2018 [3]. People who smoke are advised to consume additional vitamin C to compensate for increased requirements [7,13]. Certain disease states are associated with changes in vitamin C status. For example, the presence of certain chronic diseases (some of which result in low serum albumin concentrations) is associated with lower vitamin C status [2,14]. Acute disease can also depress vitamin C concentrations due to depletion from oxidative stress [2,15]. C-reactive protein (CRP) is a marker of acute disease and systemic inflammation [15]. It is important to include these factors in any analysis of vitamin C status using epidemiological data to control for confounding or the presence of colliders.

Multiple linear regression has been used to explore vitamin C status correlates in dietary surveys [3]. However, in biological processes, nonlinear smooth functions may better describe relationships than linear functions [16]. Particular for vitamin C, a sigmoidal relationship has already been described between vitamin C intake and status [17]; thus, nonlinear responses need to be considered. We chose to use multivariable fractional polynomials to identify important predictors for which the relationship may be nonlinear.

The aim of this analysis was to determine the effect of biological predictors on vitamin C status, with an emphasis on the effect of obesity [measured as either body weight, lean mass, fat mass, or BMI (in kg/m^2^)].

## Methods

### Study design and participants

The NHANES is a nationally representative, ongoing survey of the health and nutrition of the noninstitutionalized, civilian United States population. The aim of the survey is to provide information on the prevalence of disease, nutritional status, and for use in planning health policy [18]. Data have been collected on a continuous basis, released in 2-y cycles, since 1999. Participants are selected according to a complex, 4-stage sampling design [18]. Because some population groups are oversampled (Hispanic, non-Hispanic Black, and non-Hispanic low-income White ethnicity and income groups), the provided sample weights are used to obtain representative estimates of parameters.

The survey years 2017–2018 were chosen for this analysis due to the availability of vitamin C status data in those years. The National Center for Health Statistics Research Ethics Review Board approved protocol #2018-01 (effective beginning 26 October, 2017), continuation of protocol #2011-17 (effective through 26 October, 2017).

### Vitamin C status measurements and covariates

In NHANES years 2017–2018, vitamin C in serum was measured using isocratic ultra-HPLC, which is described in detail elsewhere [19]. The dataset was restricted to adults aged 18 y or older because normal BMI values change dynamically in children. We selected covariates for analysis based on several publications, and they were available in NHANES. Table 1 [2,3,14,15,20] describes in detail the variables chosen from the NHANES 2017–2018 survey years. We decided to focus on factors associated with an increase or decrease in vitamin C status based on a direct biological effect and did not include social or lifestyle factors that affect status indirectly through modifying dietary intake, such as nutrition knowledge or ethnicity. The covariates sex, body weight and BMI, age, cigarette smoking, physical activity, recent infection, and chronic disease were identified in a review article from Carr and Rowe as affecting vitamin C status [2]. Elevated CRP is a marker of recent infection and was chosen as a proxy for this covariate, as was a positive answer to 3 questions on recent minor illnesses in NHANES [15]. Low serum albumin is a marker of several chronic conditions, including liver disease, heart failure, and kidney disease [14].

**TABLE 1:** Summary of predictors of vitamin C status from the literature and selection of variables.

Predictor	Effect on status	Reference	NHANES dataset(s) (code)	NHANES variable name used in this analysis	Predictor description
Vitamin C status	—	—	Vitamin C (VIC_J)	LBDVICSI	Serum vitamin C measured by ultra-performance liquid chromatography
Vitamin C intake	Increase with increasing intakes	[2,3]	Dietary recall days 1 and 2 – total nutrient intake (DR1TOT_J, DR2TOT_J)	DR1TVC, DR2TVC	Vitamin C intake on dietary recall day 1 and 2
			Dietary supplement intake days 1 and 2—total nutrient intake (DS1TOT_J, DS2TOT_J)	DS1TVC, DS2TVC	Intake of vitamin C from dietary supplements on recall day 1 and 2
					Total vitamin C intakes were calculated by adding together intakes for food and supplements.
Sex	Higher in females	[2,3]	Demographics (DEMO_J)	RIAGENDR	Self-reported gender (male or female)
Age	Lower in the elderly	[2,3]	Demographics (DEMO_J)	RIDAGEYR	Age at screening
Body weight	Lower as body weight increases	[2]	Body measures (BMX_J)	BMXWT	Body weight measured in the Mobile Examination Center
BMI	Lower as BMI increases	[2,3]	Body measures (BMX_J)	BMXBMI	Calculated from weight and height measured in the Mobile Examination Center
Lean mass, fat mass	Lower as lean mass increases	[20]	DEXA (DXX_J)	DXDTOLE DXDTOPF	Total lean mass (DXDTOLE) and body fat percentage (DXDTOPF) excluding bone mineral calcium, measured by whole-body dual X-ray absorptiometry.
Acute disease	Lower after acute disease episode/when CRP is higher	[15]	HS-CRP (HSCRP_J)	LBXHSCRP	High-sensitivity C-reactive protein based on near-infrared particle immunoassay rate methodology
			Health status questionnaire (HSQ_J)	HSQ500 HSQ510 HSQ520	Question: head or chest cold last 30 d? Question: stomach or intestinal illness last 30 d? Question: flu, pneumonia or ear infections last 30 d?
Serum albumin/chronic disease	Higher when serum albumin is higher	[14]	Biochemistry profile (BIOPRO_J)	LBDSALSI	Serum albumin measured with DcX800 spectrophotometric method with bromcresol reagent
Smoking status	Lower in smokers	[2]	Smoking questionnaire (SMQ_J)	SMQ040	Question: do you now smoke cigarettes?
			Cotinine and hydroxycotinine—serum (COT_J)	LBXCOT	Serum cotinine measured by isotope-dilution HPLC
Physical activity	Increase with increasing physical activity	[2]	Physical activity questionnaire (PAQ_J)	PAD615 PAD630 PAD645 PAD660 PAD675	Minutes per day: Vigorous work-related activity (MET score 8.0) Moderate work-related activity (MET score 4.0) Walking or bicycling for transportation (MET score 4.0) Vigorous leisure-time physical activity (MET score 8.0) Moderate leisure-time physical activity (MET score 4.0) These 5 variables were converted to an overall MET score by multiplying the number of minutes per day estimated for individual activity types with the MET score for that activity type, then summing the products to produce a daily MET estimation.

Abbreviations: CRP, C-reactive protein; DEXA, dual-energy X-ray absorptiometry; HS-CRP, high-sensitivity C-reactive protein; MET, metabolic equivalent of task.

**FIGURE 1:**
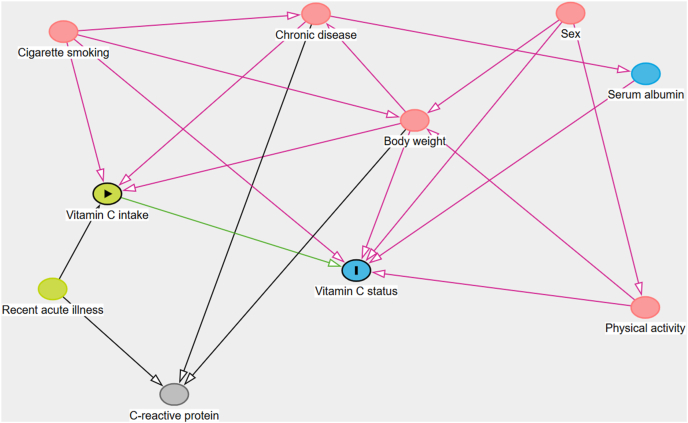
Directed acyclic graph of predictors of vitamin C status.

The demographic dataset contained information on age and gender obtained by self-report. Dietary vitamin C intake from foods was measured via 24-h dietary recall on 2 nonconsecutive days using the USDA’s Automated Multiple-Pass Method [21]. Directly after the dietary questionnaire, respondents were asked about the previous day’s intake of dietary supplements (midnight to midnight). Total supplemental vitamin C intakes were calculated by adding up the vitamin C in each supplement consumed per participant per day. Intakes from food and dietary supplements were pooled per individual because it was assumed that bioavailability is comparable. Average intake on 2 d was used as an estimate of recent vitamin C intake. Four different measures of body weight were chosen to compare the effects of total body size, adiposity, and fat-free mass: total body weight, BMI, and lean and fat mass (measured by dual-energy X-ray absorptiometry). Lean and fat mass was only available for adults up to the age of 60; therefore, they cannot be directly compared with results using the entire age range present for BMI and body weight. Higher vitamin C status is seen in physically active individuals, and the physical activity questionnaire provided an estimate of individuals’ daily metabolic activity through exercise or physical labor (Table 1 describes how the metabolic activity score was calculated). Smoking status was investigated as both a binary variable derived by self-report in a questionnaire and as a continuous variable based on serum cotinine. A directed acyclic graph was developed using the R package dagitty to assist with visualizing the relationships (Figure 1) [22].

### Statistical analysis

Data were analyzed using Stata/BE version 19.5 for Windows. Descriptive statistics were calculated using survey design parameters (Primary Sampling Units, strata) and the Mobile Examination Center 2-y sample weight to adjust for the complex sample design. Outliers in the vitamin C intake (>3000 mg/d, 3 participants), vitamin C status (>200 μmol/L, 6 participants), and physical activity (>30,000 metabolic equivalent of task, 23 participants) were removed after visual inspection of the distributions. Children aged 17 and under were removed from the dataset (3452 participants). A correlation matrix was produced using the Pearson coefficient, including Bonferroni adjustment at the 0.05 level. Multiple fractional polynomial selection was performed with the mfp command on the serum vitamin C variable using program defaults, with the exception of the body weight/BMI/lean mass/fat mass predictor selection set to 1 to force this variable into the model, as these were the variables of interest. This program uses backward elimination with the selection of a fractional polynomial function, and variables are dropped if their effect on the model is not significant according to the nominal significance level defined, which in our analysis was the default value of 0.05. Although researchers are cautioned against using this method, we include an explanatory model in Figure 1, and the dataset size is adequate, thus backward elimination within multiple fractional polynomial analysis is appropriate [23]. This model requires the outcome variable to be continuous and normally distributed, and that there are continuous model predictors. In addition, the number of covariates should be in proportion to the number of samples [24]. These assumptions were checked during the analysis process. Rows with missing values for the variables tested are not included in the analysis; thus, the number of participants per analysis varies slightly depending on the variables included. Covariates included are listed in Table 1. The Akaike Information Criterion (AIC) is reported as part of the results, which indicates the overall balance between model goodness-of-fit and simplicity, with a lower number being desirable, to allow different models to be compared. Postestimation graphs using each respective model produced were plotted using fracplot.

## Results

The characteristics of the participants are presented in Table 2 for the analysis dataset and according to BMI categories. The final overall sample size was 4556 participants. There was a greater proportion of smokers in the healthy weight category, and not surprisingly, increasingly higher body weight, fat mass, and lean mass were seen in the overweight and obese categories. Total vitamin C intake, serum vitamin C, and physical activity were lower in the obese group. CRP and recent minor illness were higher in the obese group. There was no clear difference between BMI groups for gender and serum albumin.

**TABLE 2:** Dataset characteristics.

Parameter	*N*	All		Underweight *N =* 68		Healthy weight *N =* 1096		Overweight *N =* 1436		Obese, *N =* 1956	
		Mean/percent	SE	Mean/percent	SE	Mean/percent	SE	Mean/percent	SE	Mean/percent	SE
Age, y	4556	47.9	0.659	38.3%	2.67	44.9	0.840	50.2	0.867	48.4	0.700
Gender											
Male	4556	48.8%	1.07	35.8%	8.63	42.1%	2.47	54.8%	1.69	48.8%	1.83
Female		51.2%	1.07	64.2%	8.63	57.9%	2.47	45.2%	1.69	51.2%	1.83
Serum cotinine, ng/mL	4556	55.6.7	4.87	101	27.0	70.5	7.67	46.5	4.54	52.0	5.40
BMI, kg/m^2^	4506	29.8	0.280	17.6	0.130	22.3	0.066	27.4	0.052	36.3	0.251
Body weight, kg	4513	84.6	0.804	49.5	0.970	62.8	0.360	78.1	0.373	103	0.651
Lean mass (DEXA), kg	2158	52.2	0.396	36.7	1.71	43.4	0.298	51.3	0.528	60.0	0.479
Fat mass (DEXA), kg	2073	27.7	0.556	11.9	0.705	17.5	0.230	24.8	0.284	38.2	0.622
Total vitamin C intake, mg/d	4556	165	8.02	85.0	11.6	177	17.2	179	9.73	151	9.22
Serum vitamin C, μmol/L	4556	51.1	1.34	44.1	3.74	55.4	2.21	55.9	1.11	45.4	1.37
Prevalence low serum vitamin C^1^											
Deficient	4556	6.41%	0.85	9.97%	4.5	7.46%	1.44	4.09%	0.63	7.34%	1.07
Marginal		12.6%	1.14	13.6%	5.16	10.3%	1.54	8.51%	1.57	16.8%	1.57
Low		4.21%	0.46	11.35%	9.95	2.63%	0.55	3.25%	0.77	5.55%	0.84
Adequate		79.6%	2.45	65.1%	6.63	84.2%	1.58	84.2%	1.58	70.3%	2.34
High-sensitivity C-reactive protein, mg/L	4603	3.87	0.178	6.33	5.17	2.20	0.187	2.77	0.169	5.53	0.249
Serum albumin, g/L	4612	40.9	0.169	42.0	0.631	41.7	0.265	41.3	0.171	40.1	0.167
Physical activity metabolic equivalents											
MET	4665	1106	37.3	948	185	1189	67.3	1117	59.9	1057	48.5
Recent minor illness last 30 d											
No illness	4665	78.0%	1.54	62.4%	10.0	81.2%	1.80	78.8%	1.86	76.2%	2.51
1 episode		18.6%	1.41	36.3%	10.4	15.9%	1.60	18.0%	1.90	19.9%	2.35
2 episodes		2.80%	0.34	1.31%	0.98	2.42%	0.65	2.83%	0.61	3.11%	0.54
3 episodes		0.57%	0.12	—	—	0.39%	0.27	0.38%	0.16	0.83%	0.24

Abbreviations: DEXA, dual-energy X-ray absorptiometry; MET, metabolic equivalent of task.

1 <11 μmol/L deficient; 11–23 μmol/L marginal; 23–28 μmol/L low; ≥28 μmol/L normal.

The correlation matrix is presented in Table 3. These results showed a strong positive linear correlation between body weight and BMI, and a moderate positive linear correlation between vitamin C intake and status. Negative correlations between vitamin C status and body weight, BMI, lean mass, fat mass, CRP, serum albumin, serum cotinine, and the positive correlation with age were significant but weak. No linear correlation was found between vitamin C status and physical activity.

**TABLE 3:** Correlation matrix of body weight, BMI, lean weight, and predictors of vitamin C status.

Variables	Plasma vitamin C	BMI	Body weight	Vitamin C intake	HS-CRP	Age	Serum albumin	Metabolic equivalents	Serum cotinine	Lean weight
Plasma vitamin C	1	—	—	—	—	—	—	—	—	—
BMI	−0.1589^1^	1	—	—	—	—	—	—	—	—
Body weight	−0.2083^1^	0.8915^1^	1	—	—	—	—	—	—	—
Vitamin C intake	0.4079^1^	−0.034	−0.0205	1	—	—	—	—	—	—
HS-CRP	−0.124^1^	0.2749^1^	0.2191^1^	−0.0311	1	—	—	—	—	—
Age	0.1097^1^	0.0148	−0.0412	0.1299^1^	0.0432	1	—	—	—	—
Serum albumin	0.1283^1^	–0.2818^1^	−0.1683^1^	0.0473	−0.2655^1^	−0.1708^1^	1	—	—	—
Metabolic equivalents	−0.0462	−0.0205	0.0599^1^	−0.0208	−0.0501	−0.2282^1^	0.0957^1^	1	—	—
Serum cotinine	−0.2191^1^	−0.0589^1^	0.0146	−0.0756^1^	0.0106	−0.052	−0.0423	0.1194^1^	1	—
Lean mass	−0.216^1^	0.5846^1^	0.8477^1^	−0.0143	0.0461	−0.0023	0.0484	0.1767^1^	0.1082^1^	1
Fat mass	−0.179^1^	0.918^1^	0.818^1^	−0.060	0.273^1^	0.124^1^	−0.366^1^	−0.080	−0.047	0.3925^1^

1 Pairwise Pearson correlation coefficients, values indicate significant associations at the Bonferroni-adjusted 0.05 level.

**FIGURE 2:**
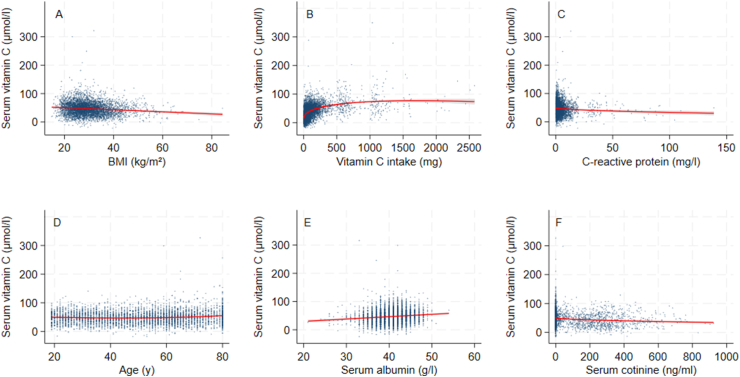
Multivariate fractional polynomial plots of serum vitamin C concentrations, BMI and other covariates from NHANES 2017–2018 (*N* = 4458). Red line reflects fitted line and gray shaded area is the 95% confidence interval for: (A) BMI, (B) vitamin C intake, (C) C-reactive protein, (D) age, (E) serum albumin, and (F) serum cotinine.

**FIGURE 3:**
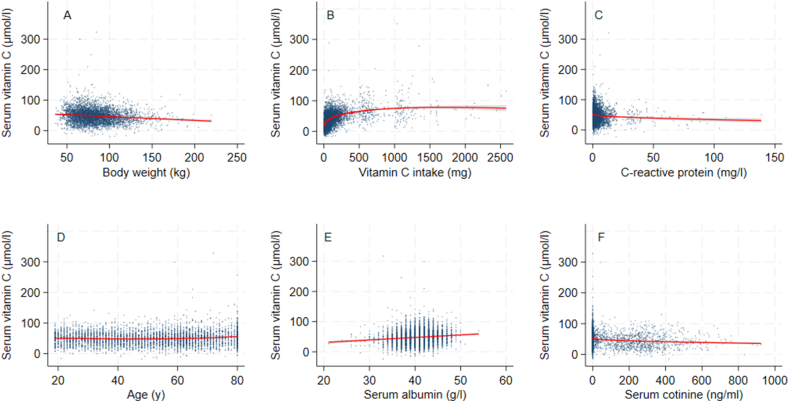
Multivariate fractional polynomial plots of serum vitamin C concentrations, body weight, and other covariates from NHANES 2017–2018 (*N* = 4464). Red line reflects fitted line and gray shaded area is the 95% confidence interval for: (A) body weight, (B) vitamin C intake, (C) C-reactive protein, (D) age, (E) serum albumin, and (F) serum cotinine.

The results of the multivariable fractional polynomial selection procedure are presented in Table 4. Margin plots of each covariate when BMI and body weight are used as measures of body size are shown in FIGURE 2, FIGURE 3, respectively. Supplemental Tables 1 and 2 and Supplemental Figures 1 and 2 show the results when lean mass and fat mass were used: due to the age cutoff for the lean mass variable of 60 y, the results cannot be directly compared with BMI and body weight; therefore, they have been included as supplemental figures and tables.

**TABLE 4:** Multivariable fractional polynomial regression model results for effect of covariates on serum vitamin C concentrations (μmol/L).

BMI (*N =* 4458, AIC = 40,570)					Body weight (*N =* 4464, AIC = 40,622)				
Parameter^1^	Power	Estimate	SE	*P* value	Parameter^1^	Power	Estimate	SE	*P* value
BMI	1	−0.365	0.052	<0.001	Body weight	1	−0.123	0.017	<0.001
Vitamin C intake, coefficient I	0.5	95.5	4.6	<0.001	Vitamin C, coefficient intake I	0.5	96.0	4.6	<0.001
Vitamin C intake, coefficient II	1	−36.5	4.0	<0.001	Vitamin C intake, coefficient II	1	−36.6	4.0	<0.001
CRP	0.5	−16.7	3.5	<0.001	CRP	0.5	−17.2	3.4	<0.001
Gender (female)	1	10.6	0.72	<0.001	Gender	1	8.92	0.76	<0.001
Age, coefficient I	2	−1.07	0.27	<0.001	Age, coefficient I	2	−0.491	0.14	<0.001
Age, coefficient II	2	0.534	0.12	<0.001	Age, coefficient II	3	0.0698	0.016	<0.001
Cotinine	0.5	−5.46	0.54	<0.001	Cotinine	0.5	−5.33	0.54	<0.001
Albumin	1	0.829	0.12	<0.001	Albumin	1	0.834	0.11	<0.001
Constant	—	46.9	7.2	<0.001	Constant	—	47.9	0.74	<0.001

Comparison of models using BMI and body weight.

Abbreviations: AIC, Akaike Information Criterion; CRP, C-reactive protein.

1 Physical activity and recent illness were removed from the final multivariable fractional polynomial model.

The multivariable fractional polynomial procedure results were similar when BMI and body weight were used. The AIC was slightly lower for BMI, indicating a marginally more parsimonious model fit. For both BMI and body weight, recent minor illness (*P* values for BMI analysis 0.925; body weight analysis 0.931) and physical activity (*P* values for BMI 0.081; body weight 0.075) were dropped; however, the other variables were retained in the model. When smoking status was included as a binary variable based on self-report rather than cotinine, physical activity was retained in the model, albeit with a very modest relationship with serum vitamin C (results not shown). BMI and body weight showed a modest inverse linear relationship with vitamin C status, with a decrease in serum vitamin C of ∼0.3 μmol/L for each unit increase in BMI and 0.1 μmol/L for each unit increase in body weight (kg). The following results apply to both fractional polynomials fitted with BMI and body weight. Vitamin C status and intake were described best by a power 2 fractional polynomial function that showed initially a strong increase in vitamin C status at low intakes, which gradually reached a plateau at between 500 and 1000 mg/d. The relationship between vitamin C status and CRP was nonlinear, with an initial strong decrease in serum vitamin C at low CRP, then a weaker decline at higher CRP concentrations. There was a weak U-shaped effect of age on vitamin C status, with slightly higher serum vitamin C in the younger and older age groups. A positive, linear relationship was found between vitamin C status and serum albumin. There was a negative, nonlinear relationship between serum vitamin C and cotinine, with an initial more rapid decrease, followed by a slight leveling as cotinine increased.

When lean or fat mass was used in the multivariable fractional polynomial selection procedure, physical activity and recent illness were also dropped (Supplemental Table 1). There was a decrease in serum vitamin C of ∼0.17 μmol/L for each unit (kilogram) increase in lean mass, or 0.33 μmol/L for each unit (kilogram) increase in fat mass. These results show that total fat mass produces a better model fit than lean mass, because the AIC is somewhat lower. Other relationships were similar to as described for body weight and BMI.

To summarize, this analysis found an association with measures of body size, age, gender, vitamin C intake, smoking status, CRP and serum albumin, whereas physical activity and self-report of recent illness were excluded. There was a negative linear relationship between vitamin C status and body size (expressed as either BMI or body weight), and a positive linear relationship between vitamin C status and serum albumin. There was a weak U-shaped relationship between age and status. A negative log function described the association between CRP and vitamin C status. For the association between vitamin C status and intake, vitamin C status increased rapidly at low intakes, then achieved a more gradual increase until attaining vitamin C status in the range of maximum body vitamin C. Vitamin C status was lower in males and smokers, with a dose-response effect when serum cotinine was used to indicate exposure to cigarette smoke (nicotine).

## Discussion

The results of this analysis are largely in line with those expected from the literature, as reported in Table 1. Overall, the effect of body size on vitamin C status was small yet noteworthy at the population level. An increase in body weight of 40 kg (the difference between the mean of the obese and healthy weight categories) was associated with a decrease in vitamin C status of 4 μmol/L. This would require an increase in vitamin C intake of ∼7 mg/d to compensate, assuming average serum vitamin C concentrations for people with obesity in our dataset of 45 μmol/L, and the dose response found in the Levine study (1.7 mg vitamin C intake/1 μM increase in serum vitamin C at this serum concentration) to raise plasma vitamin C by this amount [17]. The significantly lower vitamin C status in the obese category (but not in the healthy or overweight categories) seen in Table 1 was not reflected in the function selected by the multivariable fractional polynomial procedure, which shows a linear decline. This could be due to the inclusion of a wide range of BMIs above 30, compared with a limited range for the healthy and overweight categories.

The nonlinear relationship between intake and status is similar to what has been reported in pharmacokinetic and epidemiological studies. For example, Lykkesfeldt and Tveden-Nyborg report a steady-state at 70–80 μmol/L at a dose of 200–400 mg/d in healthy individuals [12]. Our results also show a similar plateau reached in this intake range. A series of sigmoidal curves between intake and status was developed for the cross-sectional data from the same survey years as for our analysis [25]. The shape of the curve shows the body’s efforts to conserve vitamin C by reducing excretion at low doses, whereas at high doses the active vitamin C transporters are saturated and no further increase in vitamin C status can be expected when intakes exceed a dose of around 500 mg/d.

As expected, we found lower vitamin C status in males and people exposed to nicotine: the reduction for smokers compared with nonsmokers was ∼8–9 μmol/L and for males compared with females 10–12 μmol/L, and the serum cotinine results indicate that greater exposure to cigarette smoke had a greater effect on reducing vitamin C concentrations in the blood. Another analysis using the same dataset and multiple linear regression found smokers had a similar difference to nonsmokers to what we found, whereas the effect according to gender was less pronounced (β coefficient of –5.7 μmol/L for males). The higher vitamin C intakes in males of 62 mg/d compared with females who consume 56 mg/d in this dataset [26] do not appear to be adequate to allow the vitamin C status in males to reach that of females. This would be expected because males need ∼10 mg/d more than females according to guidelines. The lower vitamin C status in smokers has been known for many decades and was taken up in United States-based dietary intake requirements, which recommend an extra 35 mg vitamin C/d [1,7]. Furthermore, smoking history may affect the relationship between vitamin C intake and status [27].

There was a weak U-shaped relationship with age, with higher vitamin C status within the younger and older age groups. The literature tends to show a decrease in vitamin C status with older age, although a recent analysis performed using the same dataset and 3 age categories also found a U-shaped relationship [27]. Potentially, older age is confounded with certain dynamic changes in body size and composition, particularly in elderly participants. For example, in elderly free-living subjects, fat mass increases at the expense of lean mass, and resting metabolic rate decreases, which may reduce the need for vitamin C in metabolic processes [28]. This may suggest that vitamin C status is affected by metabolic rate or lean mass. On the other hand, the use of medication and changes in food intake due to reduced dentition or poorer sense of smell [29] could reduce intakes of vitamin C, through lower consumption of fruit and vegetables [30]. Institutionalized elderly subjects appear at increased risk of vitamin C deficiency [31]. Ultimately, the effect of the association is rather small and whether it is found in a particular dataset may depend on how the older age group is selected and its characteristics in the survey, which may explain why the noninstitutionalized elderly subjects in this representative study showed a higher vitamin C status than younger counterparts, whereas smaller, nonrepresentative studies show a poorer status in older subjects [31]. Nonrepresentative surveys may introduce bias due to the recruitment method, such as targeting healthy elderly or those who are institutionalized or have a particular morbidity.

The relationship between physical activity and vitamin C status was not found when the smoking variable was serum cotinine; however, it was found using a binary variable based on self-report of current smoking. Potentially, information is lost when a categorical variable is used, or there may be confounding between the accurate self-report of smoking and physical activity. The relationship between vitamin C status and acute disease, reflected by either self-report of illness in the last 30 d, or an elevated CRP, was not seen in the results. However, the mild elevation in CRP, which is seen in abdominal obesity and various chronic conditions such as type 2 diabetes mellitus and cardiovascular disease [32], was associated with a lower vitamin C status. This could be due to the destruction of vitamin C due to a greater inflammatory burden.

The strengths of this analysis include the large sample size and inclusion of many potentially relevant parameters. The use of the multiple fractional polynomial selection technique allows a more sensitive analysis of associations compared with multiple linear regression, which is commonly used. For example, the coefficients found for the sigmoid relationship between vitamin C status and intake adjust for the body’s vitamin C conservation at low intakes, and saturation of absorption at high intakes, which is absent if a linear relationship is used. Most variables were analyzed as continuous data, thus avoiding artefacts introduced with categorization and maintaining power for the analysis. In addition, the presentation of margin plots allows the effect of a single variable to be visualized when other factors are held constant, aiding in the interpretation of the results.

This analysis has several limitations. First, due to the observational nature of the analysis, the associations found cannot be considered causal. We also cannot exclude the effect of confounders that were not measured or not included in the analysis. For example, an interest in a healthy lifestyle may affect several covariates in our model, such as body weight, vitamin C intake, and smoking status. This could cause confounding between several variables that cluster together. The multiple fractional polynomial method has the potential to overfit the model, such that the results cannot be generalized and are specific only to our dataset. Additionally, the measurement instruments used for each variable in the model can affect the outcome. The lack of association between recent disease and vitamin C status may be a result of the loosely formulated questions asked of participants: it is likely that recall bias, grouping of disparate diseases (e.g., “flu,” ear infection, stomach or intestinal illness in 1 question) and the length of time covered by the question of the previous 30 d may have blunted the association.

Our results confirmed several predictors of vitamin C status: vitamin C intake, body weight or BMI, gender, smoking status, serum albumin, CRP. Physical activity and recent illness were not associated with vitamin C status. Although the effect of smoking has already been taken up in dietary guidelines in some regions, more research was considered to be needed to set a recommended intake amount for smokers in others: these results support an additional requirement for smokers. The effect of BMI/body weight on vitamin C status, although assumed when extrapolating intake recommendations isometrically to females and children, has not been adequately assessed using intervention studies. The findings from this study support the isometric scaling of vitamin C recommendations based on body weight. The nonlinear relationship of covariates, including vitamin C intake, should also be taken into account. Further research in this area would be beneficial for nutrition and dietetics professionals, and for the further refinement of dietary guidelines.

## Author contributions

The authors’ responsibilities were as follows – JKB: conceived the project, developed the overall research plan, analyzed the data with analysis oversight from AM-B, and wrote the article and has responsibility for the final content; EJMF, AM-B: provided input into drafts of the manuscript; and all authors: read and approved the final manuscript.

## Data availability

The data used in this study are openly available in the NHANES website: NHANES Questionnaires, Datasets, and Related Documentation https://wwwn.cdc.gov/nchs/nhanes/Default.aspx.

## Funding

The authors reported no funding received for this study.

## Conflict of interest

JKB reports a relationship with DSM-Firmenich AG that includes: consulting or advisory, speaking and lecture fees, and travel reimbursement. Other authors declare that they have no known competing financial interests or personal relationships that could have appeared to influence the work reported in this article.
