# Supplementary material for: Predictors of Vitamin C Status in National Health and Nutrition Examination Survey 2017–2018: The Effect of Body Weight and Body Mass Index in a Multiple Fractional Polynomial Analysis
Source: J Nutr. 2025 Oct 8;155(12):4356–64. doi: 10.1016/j.tjnut.2025.10.001 (PMC12799454; doi:10.1016/j.tjnut.2025.10.001)
Supplement: Multimedia component 1 [file mmc1.pdf]

Supplemental materials

Supplemental table 1: Multivariable fractional polynomial regression model results for effect of co-variates on serum vitamin C concentrations (µmol/l) including lean mass.

Lean mass (N = 2212, AIC = 25063)				
Parameter*	Power	Estimate	Std. error	p-value
Lean mass	1	-0.00017	0.000052	0.001
Vitamin C intake coefficient I	0	13.6	0.87	<0.001
Vitamin C intake coefficient II	0	1.00	0.12	<0.001
CRP	0	-2.53	4.7	<0.001
Gender (female)	1	7.07	1.4	<0.001
Age coefficient I	3	-3.75	0.13	0.003
Age coefficient II	3	0.210	0.070	0.003
Cotinine	0.5	-5.35	0.70	<0.001
Albumin	1	0.777	0.16	<0.001
Constant	-	44.9	1.18	<0.001

*Recent illness and physical activity were removed from the final multivariable fractional polynomial model

Supplemental table 2: Multivariable fractional polynomial regression model results for effect of co-variates on serum vitamin C concentrations (µmol/l) including fat mass.

Total fat (N = 2051, AIC = 18313)				
Parameter*	Power	Estimate	Std. error	p-value
Body fat	1	-0.00033	0.000044	<0.001
Vitamin C intake coefficient I	-0.5	0.547	0.0654	<0.001
Vitamin C intake coefficient II	0	9.40	0.476	<0.001
CRP	1	-0.229	0.0717	0.001
Gender (female)	1	10.9	1.00	<0.001
Age coefficient I	3	-0.369	0.130	0.005
Age coefficient II	3	0.210	0.0719	0.003
Cotinine coefficient I	1	-5.97	0.892	<0.001
Cotinine coefficient II	2	0.704	0.178	<0.001
Serum albumin	1	0.777	0.165	<0.001
Constant	-	46.2	0.959	<0.001

*Recent illness and physical activity were removed from the final multivariable fractional polynomial model

Predictors of vitamin C status in NHANES 2017-2018: the effect of body weight and BMI in a multiple fractional polynomial analysis – Julia K. Bird

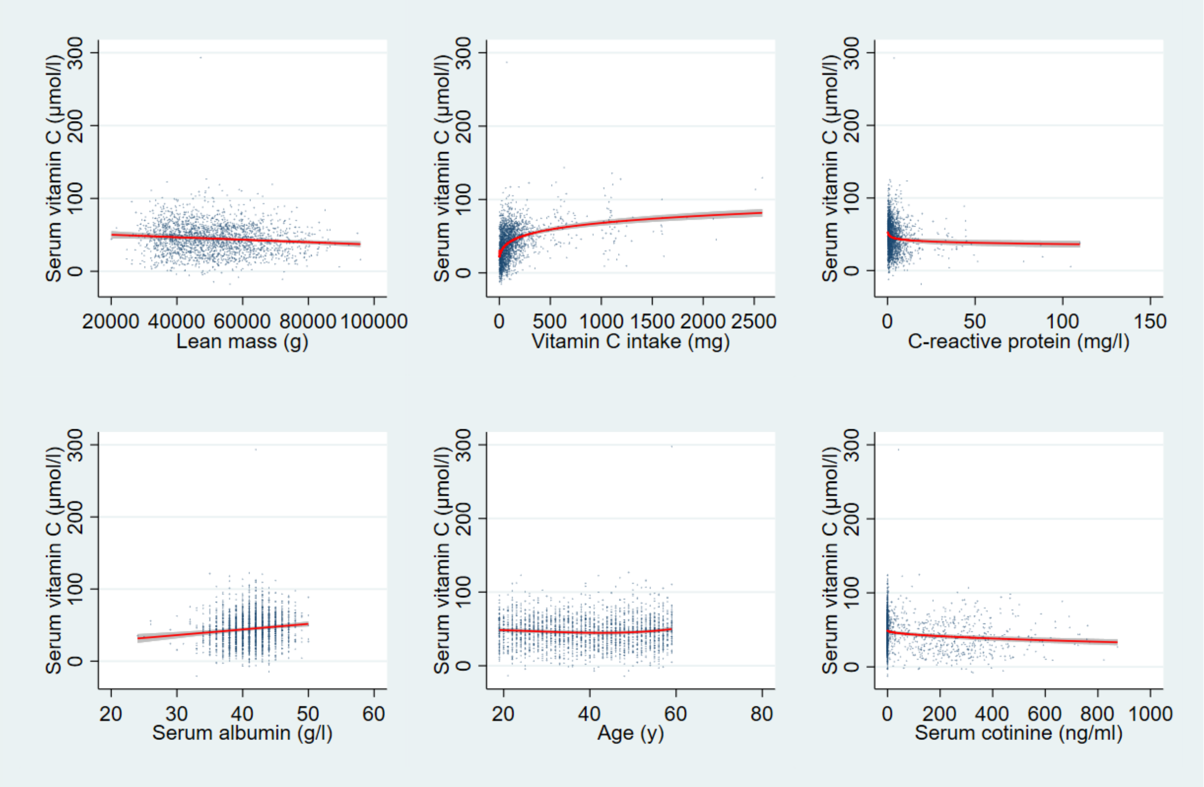

Supplemental figure 1: Margin plots of serum vitamin C concentrations, lean mass and other covariates from NHANES 2017-2018 (N=2212)

Predictors of vitamin C status in NHANES 2017-2018: the effect of body weight and BMI in a multiple fractional polynomial analysis – Julia K. Bird

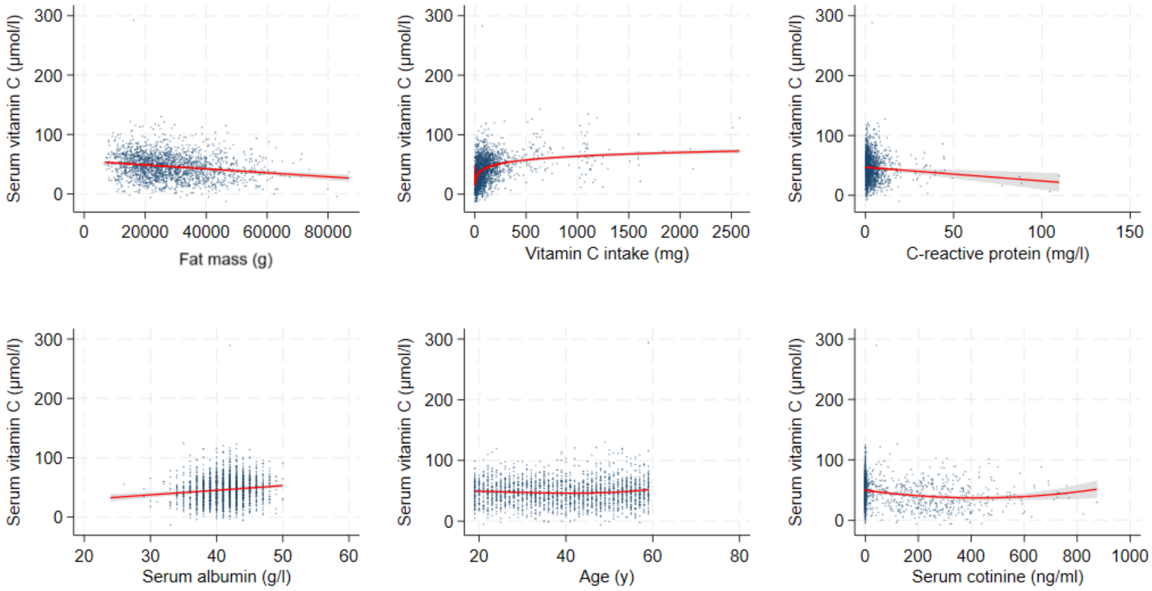

Supplemental figure 2: Margin plots of serum vitamin C concentrations, total fat mass, and other covariates from NHANES 2017-2018 (N=2212)
